# A Critical Review of Renewable Hydrogen Production Methods: Factors Affecting Their Scale-Up and Its Role in Future Energy Generation

**DOI:** 10.3390/membranes12020173

**Published:** 2022-02-01

**Authors:** Ephraim Bonah Agyekum, Christabel Nutakor, Ahmed M. Agwa, Salah Kamel

**Affiliations:** 1 Department of Nuclear and Renewable Energy, Ural Federal University Named after the First President of Russia Boris Yeltsin, 19 Mira Street, 620002 Ekaterinburg, Russia; 2 Department of Biochemistry and Forensic Science, C. K. Tedam University of Technology and Applied Sciences, Navrongo P.O. Box 24, Ghana; nutakor.christabel@gmail.com or; 3 Department of Electrical Engineering, College of Engineering, Northern Border University, Arar 1321, Saudi Arabia; 4 Prince Faisal bin Khalid bin Sultan Research Chair in Renewable Energy Studies and Applications (PFCRE), Northern Border University, Arar 1321, Saudi Arabia; 5 Electrical Engineering Department, Faculty of Engineering, Aswan University, Aswan 81542, Egypt; skamel@aswu.edu.eg

**Keywords:** hydrogen production, biomass, proton exchange membranes, alkaline electrolysis, dark fermentation, anion exchange membranes

## Abstract

An increase in human activities and population growth have significantly increased the world’s energy demands. The major source of energy for the world today is from fossil fuels, which are polluting and degrading the environment due to the emission of greenhouse gases. Hydrogen is an identified efficient energy carrier and can be obtained through renewable and non-renewable sources. An overview of renewable sources of hydrogen production which focuses on water splitting (electrolysis, thermolysis, and photolysis) and biomass (biological and thermochemical) mechanisms is presented in this study. The limitations associated with these mechanisms are discussed. The study also looks at some critical factors that hinders the scaling up of the hydrogen economy globally. Key among these factors are issues relating to the absence of a value chain for clean hydrogen, storage and transportation of hydrogen, high cost of production, lack of international standards, and risks in investment. The study ends with some future research recommendations for researchers to help enhance the technical efficiencies of some production mechanisms, and policy direction to governments to reduce investment risks in the sector to scale the hydrogen economy up.

## 1. Introduction

The increase in energy demand globally has led to an increase in discussions around clean, cheap, and sustainable sources of energy generation [1,2,3]. The global population has been estimated to hit a possible 10 billion people by 2050, which is expected to have an exponential effect on energy requirements. The use of fossil fuels as a source of energy generation has helped transform economies around the world but at a negative cost to the environment. It is for this reason, among other reasons, that researchers and scientists are investigating various forms of energy generation that comes with a zero or lower negative effect on the environment [4,5,6,7].

One of the alternatives to the conventional forms of energy generation is the use of ‘green’ hydrogen. This comes with no emissions of greenhouse gases (GHG) provided the process is done using power from renewable sources (RS). The facile electrochemical conversion, light weight, and high mass energy density of hydrogen enables it to carry energy across geographical areas in the form of liquid fuels, with freight ships or through pipelines [8]. The hydrogen atom is generally made up of a proton and an electron, and it is colorless and odorless. Its density is lower than that of air. Hydrogen’s energy gravimetric density is largely about seven times higher than that of fossil fuels [9]. Moreover, 1.0 kg of hydrogen is estimated to have a storage energy that is more than 2.75 kg that of gasoline. As a result, a liter of hydrogen has same energy as 0.25 L of gasoline [10].

As indicated above, hydrogen is produced through the conversion of materials which contain the hydrogen element, for example, carbohydrates or water. It is valued that close to 96% hydrogen worldwide is gotten from conventional fossil fuels divided as follows: 30% from naphtha reforming, 48% from steam reforming of natural gas, and 18% from coal gasification [10]. The conventional technologies used for the production of hydrogen, however, are linked to the environmental pollutions being experienced worldwide. It is for this reason that people in the energy and environment sector are pushing for more sustainable ways of producing hydrogen using RS.

**Figure 1:**
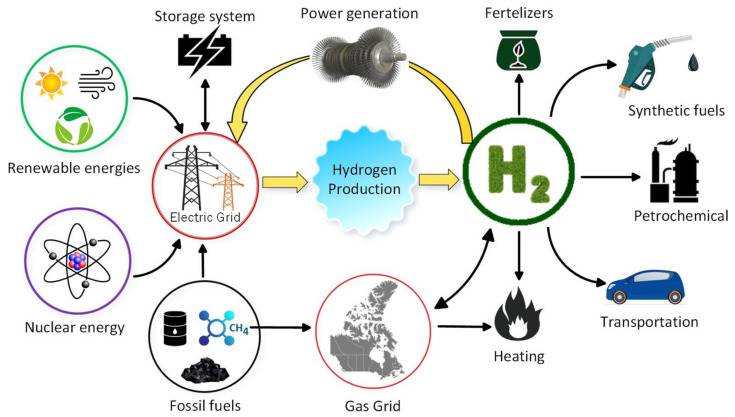
Effect of hydrogen production on other sectors [14]. Reproduced with permission from Elsevier, 2022.

Hydrogen products are currently being used as raw materials in the industrial sector. However, if we are to realize its full potential as a full energy carrier, it can play a major part in many more areas. Out of the estimated 50 million metric tons produced yearly on a global scale, its main use is as a feedstock for the production of ammonia, with oil refining taking 35% [11]. It could have major application in the freight and passenger transport (i.e., internal combustion engines and fuel cell vehicles), storage (gaseous or liquid hydrogen), thermal (natural gas blending and solid oxide fuel cells) [11,12], electricity generation and power to gas generation [13], upon realizing its role as a flexible energy carrier, etc., as shown in Figure 1.

Yue et al. [15] presented a review on recent developments of technologies on hydrogen and their application in power systems for the production of hydrogen, storage, and re-electrification. Dawood et al. [16] also reviewed various hydrogen production pathways to assess their interrelationship and interconnection on other stages of the hydrogen square. A study by Najjar [17] reviewed the safety of hydrogen in the course of production, transmission, and usage but did not look at various limitations associated with the various technologies for the production of hydrogen. According to their study, safety issues in relation to hydrogen use are mostly discussed in relation to its ignition and combustion features, i.e., low ignition energy, rapid diffusion, comparatively high flame velocity, extensive flammability range explosion level, etc. Parra et al. [18] evaluated the progress of hydrogen production from the perspective of cost; however, it lacked a detailed assessment of the various trends of the development of the various technologies. Kovac [19] reviewed hydrogen in energy transition. According to the study, this is important because the world is fast changing, which has led to discussions around technological advancement among the people. The study presented the progress being made in the hydrogen sector. Maggio et al. [20] reviewed how the production of hydrogen from renewable energy sources could affect the fuels markets. Their study suggests that producing hydrogen through electrolysis will have a significant economic impact, particularly in the area of transport, which will lead to the creation of new supply chain, and market, which will change the characteristics of the energy market. Hanley et al. [11] provided a review of hydrogen production through low-carbon methods from varying integrated energy system models. Their objective was to identify the policy scenarios and factors that lead to the advent of hydrogen over other low carbon technologies. They identified that bioenergy could serve as both a driver and competitor for hydrogen energy, along with high renewable electricity scenarios and increased electrification. Electric vehicles were, however, identified as the key competitor in the passenger vehicle sector. Additionally, Abe et al. [21] presented a short overview of hydrogen as an energy carrier. The study identified the storage of hydrogen as a hinderance to its development and provided some recommendations for its development.

Finally, Liu et al. [22] looked at the trends and future challenges for the production and storage of hydrogen. According to their findings, the most researched hydrogen production mechanism during their study period, i.e., 2004–2018, is the photocatalytic decomposition of water to hydrogen. Mengdi and Wang [23] recently provided an overview of technologies use for the production of hydrogen, which includes both renewable and non-renewable resources. They also compared the life cycle environmental impact assessment for the various technologies. Hosseini and Wahid. [24] also looked at various hydrogen production technologies. Their findings indicate that the high cost of photovoltaic cells and their low efficiency are the most critical barriers for the commercialization of solar based hydrogen production. Similarly, El-Emam and Ozcan [25] provided an analysis on the economic, technological, and environmental aspects of hydrogen production. They observed that the lower cost of electricity that is associated with geothermal and nuclear energies makes them the ideal sources for the production of hydrogen at low-cost. Okonkwo et al. [26] conducted a study on the possibility of producing, using and exporting carbon-free hydrogen from Qatar. The findings of their paper shows that the best pathway for Qatar at present is the production as well as exportation of blue ammonia, whereas green hydrogen could in the mid-future become as competitive as blue ammonia. In another study, Lane et al. [27] forecasted renewable production of hydrogen technology shares. The outcome of the study suggests that the dominant technology consists of biomass gasifiers on the early market; however, electrolyzers’ higher learning rate as well as the long-term trend of declining cost for RE could lead to equal shares for installations that are new by mid-term and ultimately to electrolyzers having the leading share of new facilities.

In this paper, a review of clean hydrogen production technologies is presented. This is not the first time the different hydrogen production technologies have been discussed, as there have been some studies, discussed in previous sections, and some of these [28,29,30,31] have reviewed one or more forms of the processes associated to hydrogen production. Aside from the emphasis on clean technologies in this paper, it also moves beyond the review of the technologies alone and presents the limitations associated with them. Challenges in the sector that is hindering the progress of the hydrogen economy globally are also presented. The role of hydrogen in the world’s energy sector is also presented.

## 2. Status of the Global Hydrogen Production

**Figure 2:**
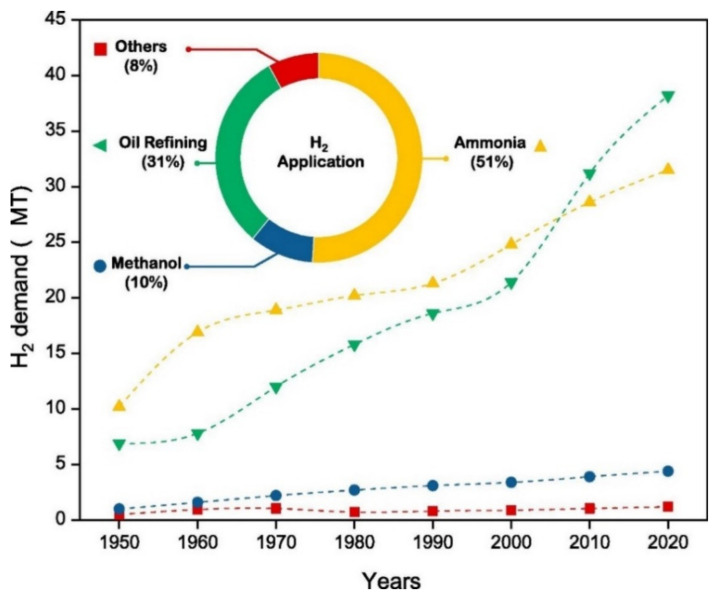
Demand of hydrogen worldwide and its share on various uses [35]. Reproduced with permission from Elsevier, 2022.

According to the International Energy Agency (IEA) [32], technologies for the production of hydrogen were unusually robust during the COVID-19 pandemic, with their momentum in 2020 remaining strong. According to the IEA report, the year 2020 was a record year in policy action and low-carbon production, where a total of ten governments across the globe adopted hydrogen strategies. An installation of about 70 MW capacity of electrolysis was done, which doubled the preceding year’s record, and two hydrogen producing facilities from fossil fuels with Carbon Capture, Utilization, and Storage (CCUS) became operational, which led to about a 15% increase in production capacity [32]. Reports show that the global total hydrogen demand has increased rapidly by 27.2% in 7 years, i.e., from 255.3 billion cubic meters in 2013 to some 324.8 billion cubic meters in 2020 [33,34]. Figure 2 shows the increase in demand for H_2_ over the years; it can be seen that the produced hydrogen are mostly used in the production of ammonia (51%), while about 31% goes into oil refining, 10% is used for the production of methanol, and the remaining 8% has other uses [35].

The hydrocarbon steam reforming is presently the most utilized approach for the production of hydrogen globally, it is used in over 90% of industrial hydrogen production facilities. It is a technology that was invented in 1926 by the Badische Anilin-und-Soda-Fabrik (BASF) [34,36].

Reports by the Hydrogen Council and McKinsey & Company [37] indicated that out of the 228 large-scale industrial, infrastructure, and transport hydrogen projects around the world, more than half, i.e., 126, are projected to be sited in Europe, 19 in North America, 24 in the Oceania, and 46 in Asia. These projects are estimated to be a potential investment of 1.4% of the global energy fund, which is equivalent to $300 billion. According to the same report, a total of 75 countries around the world, which translates into more than half of the world’s gross domestic product (GDP), have instituted net-zero carbon ambitions and over 30 have strategies directed at hydrogen production.

## 3. Paths to Hydrogen Production

**Figure 3:**
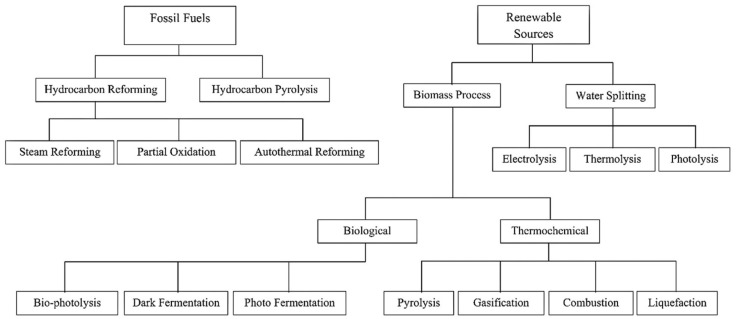
Routes to hydrogen production [28,40]. Reproduced with permission from Elsevier, 2022.

Production of hydrogen is mainly done using either fossil fuels or through RS, which is presented in Figure 3. The widely used approach is called “steam reforming”. Methane is the widely used fuel in this process due to its high hydrogen-to-carbon ratio within the hydrocarbons group; therefore, the generated by-products are reduced [38]. The steam methane reforming (SMR) process is generally made up of two steps, as presented below [39]:

- The initial step is the reformation process; at this stage, there is the mixing of the methane with steam, which is moved over a catalyst bed with a high pressure of 1.5–3 MPa and a temperature range of 700–900 °C to form a combination of carbon monoxide (CO) and hydrogen, as presented in Equation (1).
- The next phase involves the shift reaction, where there is the reaction of additional steam with the CO from the initial phase to produce additional hydrogen and CO_2_, as indicated in Equation (2):
  (1) $${\mathrm{CH}}_{4}+H_{2}O\to \mathrm{CO}+3H_{2}$$
  (2) $$\mathrm{CO}+H_{2}O\to {\mathrm{CO}}_{2}+H_{2}$$

Coal gasification is the other method for the generation of hydrogen that uses fossil fuels. In this method, the coal is taken through a partial oxidation at high pressure, approximately 5 MPa, and temperature with the assistance of steam and oxygen to yield a combination of CO, methane, CO_2_, and other compounds [41]. Hydrogen and CO mostly remain at temperatures beyond 1000 °C and pressures of 1 bar. The process is presented in Equations (3) and (4) [39]:

(3) C+12O2→CO

(4) $$C+H_{2}O\to \mathrm{CO}+H_{2}$$

This study, however, only focuses on the various renewable (RE) options and discusses them in detail in subsequent sections below. A review of recent studies using different methodologies are presented in Table 1.

### 3.1. Water Splitting

Water is seen as the most abundant source for the production of hydrogen, which is made up of hydrogen and oxygen. Therefore, if sufficient energy is given to it, its molecules will split into hydrogen and oxygen. Several technologies can be adopted to split the water. Some of these technologies are discussed in detail in the subsections below.

#### 3.1.1. Electrolysis of Water for the Production of Hydrogen

In this process, the reactant is water, which is dissociated into oxygen and hydrogen using DC current:

(5) $$\mathrm{Anode}:{\;H}_{2}O\to 1/2O_{2}+2H^{+}+2e^{-}$$

(6) $$\mathrm{Cathode}:2H^{+}+2e^{-}\to H_{2}$$

(7) $$\mathrm{Overall}:{\;H}_{2}O\to H_{2}+1/2O_{2}$$

There are different electrolyte systems that can be used for water electrolysis, which includes anion exchange membranes (AEMs) electrolysis, alkaline water electrolysis (AWE), solid oxide water electrolysis (SOE), and proton exchange membranes (PEMs) electrolysis. They operate using same principle, although they operate under different conditions using different materials [51].

##### Alkaline Water Electrolysis

This method makes use of two non-platinum group metals, i.e., nickel (Ni) and iron (Fe) based electrodes, 30–40% potassium hydroxide (KOH) electrolyte, and a diaphragm membrane. Alkaline electrolysis is seen as a technology that is reliable for hydrogen production up to the megawatt scale [52,53]. The alkalinity is provided by the circulating KOH electrolyte. The permeable diaphragm plays the role of a separator to separate the cathode and the anode. It also plays the following roles: conduction of hydroxyl ions, ensuring safety and efficiency, and prevention of possible gas crossover [52,53,54]. Organic polymers, such as polypropylene, as well as ceramic oxide materials, e.g., potassium titanate and asbestos, can be used for the construction of the diaphragm [55].

A typical AWE works at a current density that ranges from 300–400 mA cm^−2^, with a cell voltage that ranges from 1.85–2.2 V, moderate temperature of 70–90 °C, and conversion efficiencies ranging from 60–80%. The merits of this technology are that it is independent of a noble metal catalyst for the production of hydrogen, and its relatively low temperature makes its handling easier [56].

##### Solid Oxide Water Electrolysis

This is an innovative concept which allows water or steam electrolysis at temperatures that range from 600–900 °C. Higher efficiencies are obtained in this approach compared to the PEM and alkaline electrolyzers. Both recycled hydrogen and steam are supplied to the cathode; water is then reduced to yield hydrogen, as indicated in Equation (8). The oxide anions produced at the cathode goes through the solid electrolyte to the anode, and at this stage, they recombine to form oxygen and closes the circuit with the electrons released, as indicated in Equation (9) [52]:

(8) $$H_{2}O\left(g\right)+2e^{-}\to H_{2}\left(g\right)+O^{2-}$$

(9) $$O^{2-}\to \frac{1}{2}O_{2}\left(g\right)+2e^{-}$$

**Figure 4:**
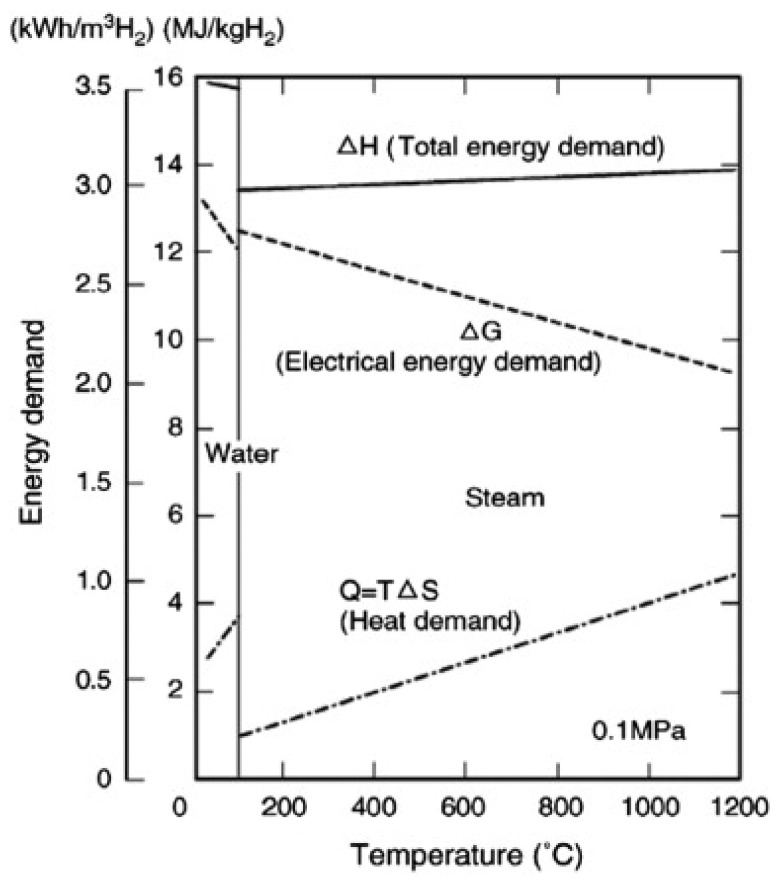
Water and steam electrolysis energy demand [57]. Reproduced with permission from Elsevier, 2022.

In this concept, the reaction changes with the electrodes in contact with the vapor phase or gas, which is a clear deviation from the processes that takes place on the electrodes of the PEM or alkaline electrolyzers. This makes it challenging to maximize the interfacial area which is in contact between the gaseous chemical species and electrodes [52]. Figure 4 shows the water and steam electrolysis energy demand.

##### Proton Exchange Membranes

PEM is mostly employed in fuel cells for electricity generation and in electrolyzers for hydrogen production. It also serves as a separator between the cathode and the anode. The most used PEMs are the Nafion™ and Nafion™-based membranes as a result of their high thermostability, high ionic conductivity, excellent chemical stability, good mechanical strength, and robustness at a low temperature during high levels of relative humidity [58]. There are, however, some major challenges associated with the use of the Nafion™, i.e., poor proton conductivity when temperatures are high under low humid environment and longer time needed for synthesis [58,59,60].

In PEM electrolyzers, there is the introduction of water at the anode where splitting into proton and oxygen occurs. The proton then moves to the cathode through the membrane, where recombination occurs to form hydrogen [61]. The oxygen gas is left behind with the unreacted water. A drier may be employed to eliminate residual water depending on the requirements for purity after the liquid/gas separations unit. There is a low ionic resistance in PEM electrolyzers; hence, a high current greater than 1600 mA cm^2^ can be attained, while high efficiencies of 55–70% is maintained.

The cathode and anode reactions are presented below [61]:

(10) $$\mathrm{Anode}:\;2H_{2}O\to O_{2}+4H^{+}+4e^{-}\;$$

(11) $$\mathrm{Cathode}:4H^{+}+4e^{-}\to 2H_{2}$$

(12) $$\mathrm{Overall}:H_{2}O\to H_{2}+\frac{1}{2}O_{2}\;\Delta H=-288{\mathrm{kJmol}}^{-1}$$

##### Anion Exchange Membranes

**Figure 5:**
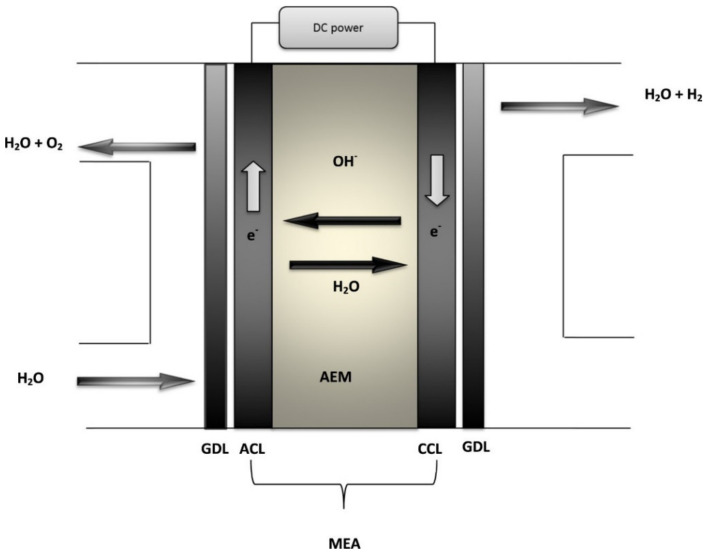
Diagram of an AEM water electrolysis. CCL: cathode catalyst layer, ACL: anode catalyst layer, GDL: gas diffusion layer, and MEA: membrane electrode assembly [56]. Reproduced with permission from Elsevier, 2022.

The AEM method is a hybrid technique (i.e., in relation to water electrolysis), which integrates the merit of alkaline and PEM electrolysis within a cell, which consists of an AEM that is hydrocarbon-based and two transition metals, for example platinum (Pt), iridium (Ir), etc., of catalyst-based electrodes [62]. Figure 5 shows the process of AEM and the components involved. There is a connection of an external power supply to the cathode and anode for the provision of DC current. The entire reaction is made up of two half-cell reactions, i.e., oxygen evolution reaction (OER) and the hydrogen evolution reaction (HER). Water is passed through the side of the anode, which produces hydrogen and hydroxyl ions through the adding of two electrons. Then, there is the diffusion of the hydroxyl ions through the AEM to the portion of the anode through the positive attraction of the anode, whereas through the external circuit the electrons move to the anode. The hydroxyl ions recombine in the anode chamber as oxygen and water through the loss of electrons. The oxygen is released from the anode’s surface by forming bubbles. Catalytic activity is required by both half-cell reactions for the formation and emission of the corresponding gases from the surfaces of the electrode. The half-cell reactions in Equations (13)–(15) apply [56,63]:

(13) $$\mathrm{Anode}:4{\mathrm{OH}}^{-}\to O_{2}+2H_{2}O+4e^{-}\;{\;E}_{0}=0.401\;V.$$

(14) $$\mathrm{Cathode}:4H_{2}O+4e^{-}\to 2H_{2}+4{\mathrm{OH}}^{-}\;{\;E}_{0}=-0.828\;V$$

(15) $$\mathrm{Overall}:2H_{2}O\to 2H_{2}+O_{2}\;{\;E}_{0}=1.23\;V$$

The reaction needs a theoretical thermodynamic cell voltage of 1.23 V at a temperature of 25 °C in order to dissociate the water into hydrogen and oxygen. However, in practice, in order to obtain an efficient hydrogen production, the cell voltage must be greater than 1.23 V. Extra voltage is needed to overcome the ohmic resistance and the kinetics of the electrolyte as well as the electrolyzer’s components [56].

#### 3.1.2. Thermolysis of Water for Production of Hydrogen

The thermolysis of water, which is referred to as a single stage thermal dissociation of water, is presented as follows [64]:

(16) $$H_{2}O\overset{\mathrm{heat}}{\to }H_{2}+\frac{1}{2}O_{2}$$

The reaction in this approach requires a source of heat to be able to get a reasonable level of dissociation; this heat source should be able to make available temperatures beyond 2500 K. For example, the level of dissociation at 3000 K and 1 bar is 64% [64]. These are the main challenges associated with this method of production. Another challenge associated with this form of hydrogen production is the high cost of its equipment due to the need to withstand the high conditions presented earlier in this section. Different technologies are, therefore, being proposed in order to conduct thermolysis at lesser temperatures. One of such strategies involves the conduction of the reaction in a number of phases with the use of catalysts; however, the requirement of very corrosive reagents also poses challenges to equipment as well as a possible negative impact on the environment [65].

Kasai and Bishop [66] proposed a single reaction system, which can realize an entropic exchange for the production of hydrogen through thermolysis by means of zeolites. Free hydrogen as well as oxidize bivalent cations are produced from the rehydration of zeolites, which happens at temperatures around 400 °C. In the same way, Hsu [67] proposed a raney nickel plate reactor (10% Aluminum and 90% Nickel), which functions as a catalytic surface to change the reaction’s energy activation. Additionally, iodine ($I_{2})$ and sulfur dioxide (${\mathrm{SO}}_{2})$ were added to the medium, which had a decreasing effect on the temperature of the reaction, as presented in Equations (17) and (18) [65]:

(17) $$I_{2}+{\mathrm{SO}}_{2}+2H_{2}O\to 2{\mathrm{HI}}_{\left(I\right)}+H_{2}{\mathrm{SO}}_{4\left(I\right)}.\;$$

(18) $$2{\mathrm{HI}}_{\left(I\right)}\to H_{2\left(g\right)}+I_{2\left(I\right)}\left(200\approx 400\;{}^{\circ}C\right)$$

#### 3.1.3. Photolysis of Water for Hydrogen Production

Water can release hydrogen in principle, which occurs when the water molecules absorb energy at a rate of 285.57 kJ/mole of water from ultraviolet radiation [68]. The dissociation of the H-O bonds by photons is known as photolysis, which occurs at about 190 nm [69]. This process and thermolysis need chemical catalysts such as ZrO_2_, tin oxide (SnO), zinc oxide (ZnO), and other semiconductor sulfur oxides [65] (Equations (19) and (20)):

(19) $${\mathrm{Mox}}^{Z+}+H_{2}O\to {\mathrm{Mox}}^{\left(Z+1\right)}+H+{\mathrm{OH}}^{-}$$

(20) $$2H\to H_{2}$$

#### 3.1.4. Some Limitations Associated with Water Electrolysis Technologies

Some limitations associated with some of the technologies presented above are presented below [70]:

- Solid oxide electrolysis—requires a relatively large laboratory stage, low durability, and large system design.
- PEM electrolysis—has an acid environment, immature and costly components, and low durability.
- Alkaline electrolysis—has low current densities, purity of gases is low, low dynamic operation, operational pressure is low (3–30 bar), and there is a reduction in the electrolyzers performance due to the formation of carbonates on the electrode.

### 3.2. Biomass Process of Hydrogen Production

**Figure 6:**
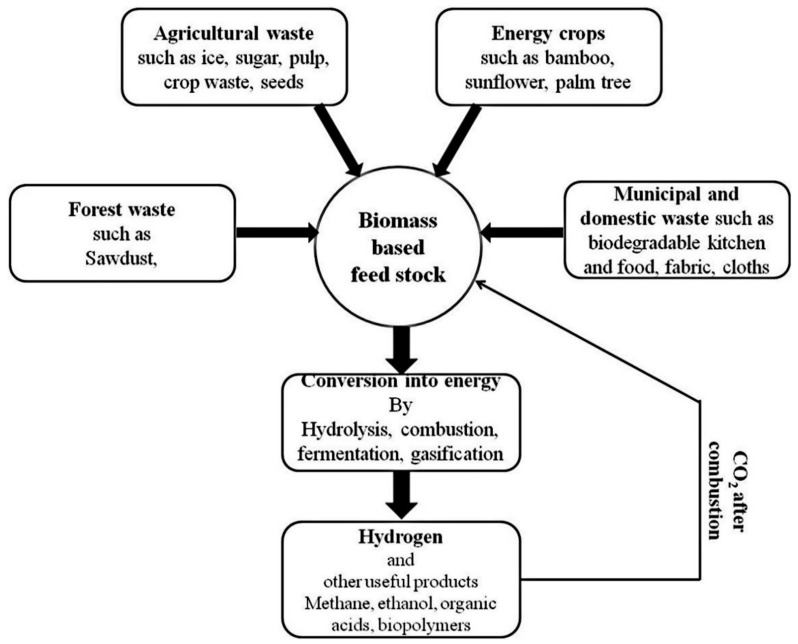
Sources of biomass and their conversion into hydrogen and other beneficial products [72]. Reproduced with permission from Elsevier, 2022.

Biomass is seen as more promising compared to fossil fuels in terms of hydrogen production due to its large reserves and supply, easy oxidation, and high annual output. Therefore, hydrogen can be produced in various forms in relation to biomass as shown in Figure 6. This includes thermochemical conversion of wood waste, photocatalysis (PC) of municipal solid waste, lignin, sawdust, forest residues, agricultural waste, cellulose, polyols, fermentation of microalgae and cassava, biomethane (biogas), steam reforming of gasified biomass tar, etc. [34]. Even though there is a release of CO_2_ during the production of hydrogen using biomass, the quantity of gaseous emissions is equal to the quantity absorbed by organisms during their lifetime [71]. Biological and thermochemical mechanisms are the two approaches that can be used to produce hydrogen from biomass. These will be discussed in subsequent sections.

Different from coal, biomass has a comparatively high hydrogen-to-carbon ratio. It is estimated that C_6_H_10_O_5_ (pure cellulose) has a hydro-carbon ratio of approximately 1.7 as compared to that of 0.8 for a characteristic bituminous coal [73]. Using biomass can decrease the dependance on hydrocarbons. Biomass can fix CO_2_ balance in the atmosphere through a mechanism known as photosynthesis process [74].

#### 3.2.1. Biological Production

##### Dark Fermentation Process of Hydrogen Production

Dark fermentation (DF) is seen as the most promising technique for the generation of biohydrogen via the conversion of biomass, it has a net energy ratio equivalent to 1.9, while that of steam methane reforming is equal to 0.64 [75]. Production of hydrogen can be done by anaerobic bacteria, which are grown in carbohydrate rich or dark substrate. The anaerobic metabolism of pyruvate, which is formed due to the catabolism of various substrates, drives most microbial hydrogen production. One of two enzyme systems catalyze pyruvate breakdown [75]:

1. *Pyruvate: formate lyase:*
  (21) Pyruvate+CoA→acetyl−CoA+formate
2. *Pyruvate: ferredoxin oxido reductase:*
  (22) $$\mathrm{Pyruvate}+\mathrm{CoA}+2\mathrm{Fd}\left(\mathrm{ox}\right)\to \mathrm{acetyl}-\mathrm{CoA}+{\mathrm{CO}}_{2}+2\mathrm{Fd}\left(\mathrm{red}\right)$$

**Figure 7:**
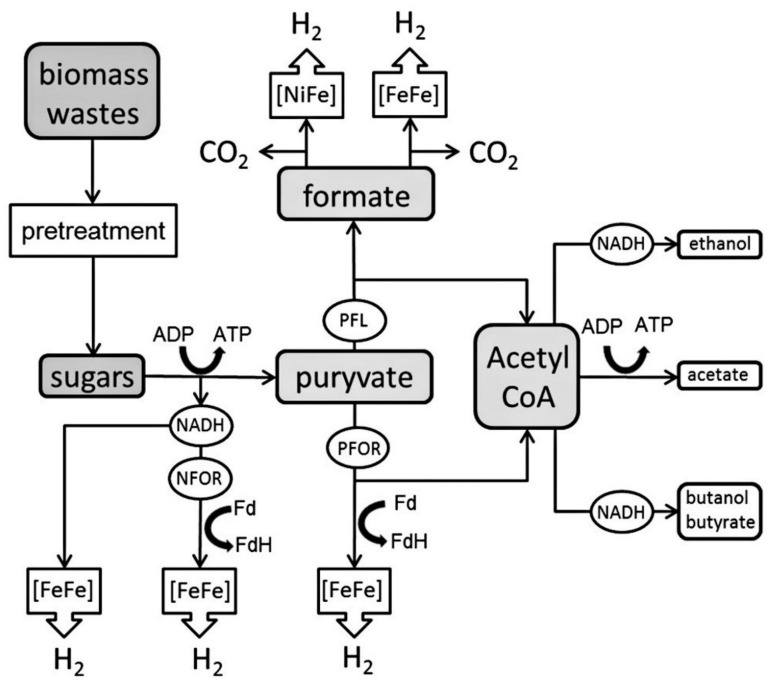
Characteristic metabolic ways for converting substrate to hydrogen during DF [76]. Reproduced with permission from Elsevier, 2022.

Substrates in DF are transformed by anaerobic bacteria grown in the dark (Figure 7). The key substrate in metabolism is hydrogen for several anaerobic microorganisms. If they are available, such microorganisms are capable of utilizing gydroenergy-rich hydrogen molecules, to produce energy through the use of the electrons from the hydrogen oxidation. When there are no external acceptors of electrons, the organisms have extra generated electrons in the process of metabolism due to the reduction of protons producing hydrogen molecules. Hydrogenases are the key enzymes that regulate hydrogen metabolism [76].

##### Photo Fermentation Process of Hydrogen Production

Photo fermentation is considered as one of the probable pathways for the production of biological hydrogen. Anoxygenic photosynthetic bacteria, especially the purple non-sulfur (PNS) bacteria, are in this process able to reduce H^+^ ions to gaseous H_2_, through the use of both the reduction power obtained from the organic compounds’ oxidation, e.g., low-molecular weight fatty acids as well as light energy. This mechanism is regarded as promising as a result of the absence of O_2_-evolving reactions, the potential to utilize varied range of sunlight, high substrate conversion yields, and the potential to combine this form of H_2_ production methods with waste disposal [77].

**Figure 8:**
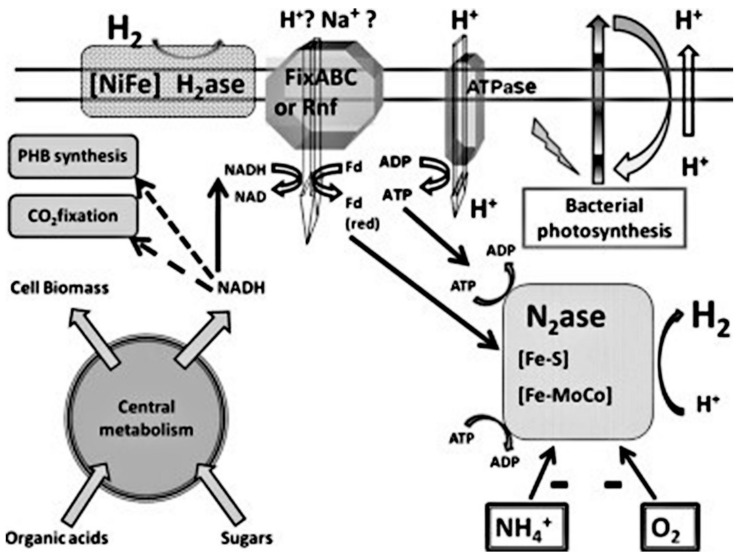
Production of hydrogen via photo-fermentation through photosynthetic bacteria paths, resulting in the production of hydrogen via a non-sulfur-deprived photosynthetic bacterium [78]. Reproduced with permission from Elsevier, 2022.

Rhodobacter, a PNS bacterial genus, is the most commonly used for the production of biohydrogen. PNS bacteria can grow photo-heterotrophically and acquire their carbon needs and electrons from reduced fixed carbon compounds, using CO_2_ as the only source of carbon and H_2_, S_2_, or Fe^2+^ as electron donors, while some species can also grow photo-lithoautotrophically. The PNS can utilize a variety of organic carbon compounds: amino acids, alcohols and carbohydrates, acetate, and other organic acids and pyruvate depending on the species. Other species can also utilize compounds with a one-carbon atom, such as formate and methanol, whereas others grow with the use of aromatic organic compounds, e.g., chlorobenzoate, cinnamate, benzoate, phenol, and phenylacetate [78]. Figure 8 shows the production of hydrogen through photo-fermentation.

The reaction for the production of hydrogen using photo fermentative mechanism using acetate is presented in Equation (23). The reaction is not spontaneous due to its positive free energy and as a result requires external energy in the form of light (natural or artificial). Four moles of hydrogen can be theoretically produced from the 1 mol of acetate under adequate physico-chemical conditions [79].

(23) $$2{\mathrm{CH}}_{3}\mathrm{COOH}+2H_{2}O\to 4H_{2}+2{\mathrm{CO}}_{2},\;\Delta \mathrm{Go}=+104\;\mathrm{kJ}$$

##### Bio-Photolysis Process of Hydrogen Production

This is a biological process that employs similar principles that are seen in algae and plants photosynthesis which are adapted for the production of hydrogen gas. Only carbon dioxide reduction occurs in green plants, as there is an absence of the enzyme that catalyze the formation of hydrogen. However, there exist hydrogen-producing enzymes in algae which can produce hydrogen under certain situations [80]. Bio-photolysis can take place in either directly or indirectly form under illumination, which then results in H_2_ and O_2_ production [81].

**Figure 9:**
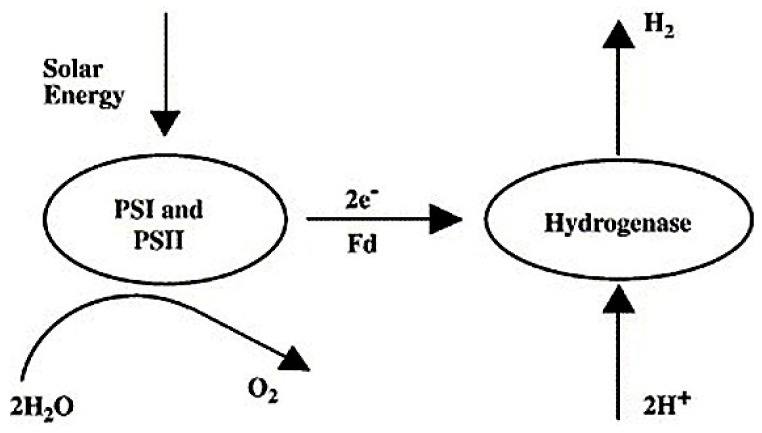
Representation of direct bio-photolysis [83]. Reproduced with permission from Elsevier, 2022.

The most analyzed species for direct bio-photolysis is the microalgae chlamydomonas reinhardtii. Both photosystems (PSI and PSII) and hydrogenase enzymes play active roles in direct bio-photolysis [82]. The molecules of water are split by green algae in direct bio-photolysis to form oxygen and hydrogen ion via photosynthesis. H_2_ is produced through the conversion of the generated hydrogen ions by hydrogenase enzyme. This enzyme is extremely sensitive to oxygen, and as a result, the oxygen content must be maintained below 0.1%, which is a drawback of this technology [83]. The process of direct bio-photolysis is presented Figure 9.

**Figure 10:**
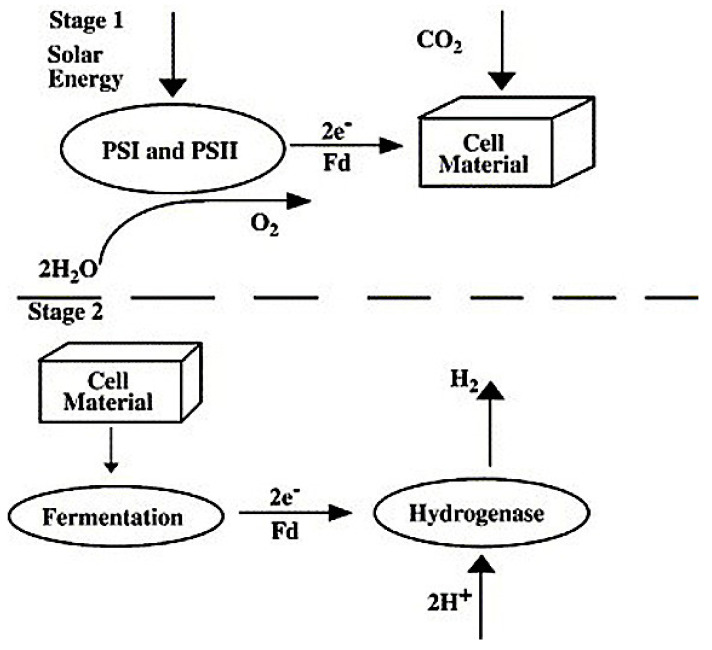
Production of hydrogen using an indirect bio-photolysis approach [83]. Reproduced with permission from Elsevier, 2022.

Indirect bio-photolysis could be used with many of the cyanobacteria. In this case, the process of the photosynthesis is accompanied by dark fermentation to produce H_2_O and H_2_. The conversion of CO_2_ into an endogenous reserve carbohydrate occurs before the production of hydrogen in the presence of hydrogenase enzyme [82,84]. Figure 10 illustrates the indirect mechanism of the production of hydrogen. The indirect bio-photolysis concept can be divided into four groups [83]:

- Production of biomass via photosynthesis.
- Biomass concentration.
- Aerobic DF producing 4 mol hydrogen/mol glucose in algae cell, accompanied by 2 mol of acetates.
- Production of hydrogen through the conversion of 2 mol of acetates.

*Cyanobacteria* are used in a typical indirect bio-photolysis to produce hydrogen through the reactions provided in Equations (24) and (25):

(24) $$12H_{2}O+6{\mathrm{CO}}_{2}\to C_{6}H_{12}O_{6}+6O_{2}$$

(25) $$C_{6}H_{12}O_{6}+12H_{2}O\to 12H_{2}+6{\mathrm{CO}}_{2}$$

#### 3.2.2. Thermochemical Production

The thermochemical method is a system whereby biomass is converted into hydrogen rich gases and hydrogen. The production of hydrogen-rich gases from synthesis gas obtained from such methods is seen as the way forward for zero GHG emissions, which is necessary for sustainable development [28,85]. The thermochemical method is mainly made up of gasification and pyrolysis. The two conversion methods produce CH_4_ and CO, among other gaseous products, which can be treated more for the production of additional hydrogen through water gas shift (WGS) and steam reforming reaction. Liquefaction and combustion methods are additional mechanisms that are two less preferable approaches, since they produce low hydrogen with the release of byproducts and also requiring operating conditions of 5–20 MPa without the presence of air, which are difficult to meet [83].

##### Pyrolysis Way of Hydrogen Production

Pyrolysis or co-pyrolysis is another promising technique for the production of hydrogen. In this technique, heating and gasification of raw organic material occur in a temperature range of 500–900 °C at a pressure of 0.1–0.5 MPa [86,87]. The process happens in the absence of air and oxygen, and consequently, the formation of dioxins can be ruled out. The absence of air or water means there will be no formation of carbon oxides (CO_2_ and CO) to require secondary reactors. As a result, this process of hydrogen production helps to reduce emissions. However, in the situation where there is the presence of water or air (i.e., the materials are not dried), there will be significant emission of CO*_x_*. Some of the merits of this technology are its clean carbon byproduct, fuel flexibility, reduction in CO*_x_* emissions, and comparative simplicity and compactness. The reaction for this mechanism is presented in Equation (26) [87]:

(26) $$C_{n}H_{m}+\mathrm{heat}\to \mathrm{nC}+0.5{\mathrm{mH}}_{2}$$

Pyrolysis can be grouped into high (over 800 ℃), medium (500–800 ℃), and low temperatures (up to 500 ℃). Fast pyrolysis (FP) is a process that is used to transform organic material into products with higher energy content. Products of FP appear in all three phases, i.e., liquid, solid, and gas. The possibility for fouling by the formed carbon is one of the challenges associated with this method; however, its proponents are of the view that it can be reduced using the suitable design [88]. Pyrolysis could play a key role in the production of hydrogen, since it has the ability to reduce the emission of carbon monoxide and carbon dioxide and could also be operated in a way that recovers a significant portion of solid carbon [87].

##### Hydrogen Production through Gasification

Biomass gasification is regarded as a potential way of RE hydrogen production. Biomass gasification is identified as the most economical and efficient way for the production of hydrogen. It is a high temperature partial oxidation process where biomass, which is a solid carbonaceous feedstock, is transformed into a gaseous mixture (CH_4_, CO_2_, H_2_, CO, tar, light hydrocarbons, ash, minor contaminates, and char) using gasifying agents [89]. Biomass gasification’s performance is affected by at least 20 operational parameters regarding the feedstock and gasifier, which include the gasifier’s geometrical configuration, flow rate, type of gasifying agent, reaction/residence time, pressure and temperature of the gasification, the gasifying agent/biomass ratios, etc. [89,90]. The product distribution and the composition of gas hinges on several factors, which include the reactor type and the temperature of the gasification. The most vital gasifier types are the entrained flow gasifier and the fixed bed gasifier (updraft or downdraft fixed beds). A significant gas conditioning needs to be included in these gasifiers along with inorganic and tars impurities removal and the subsequent transformation of CO to H_2_ through WGS [91].

**Figure 11:**
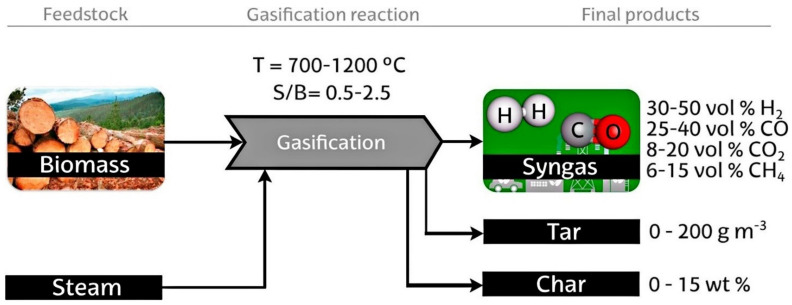
Diagram of the biomass steam gasification procedure for hydrogen production [92]. Reproduced with permission from Elsevier, 2022.

Biomass gasification normally occurs withing a temperature range of 700–1200 ℃, using oxygen, air, steam, or their combination as a gasifying agent. Steam enhances the formation of H_2_ and generates a high heating value gas without nitrogen. Even though steam gasification has higher energy cost as a result of its highly endothermic nature compared to air gasification, it prevents the need for an expensive oxygen separation process [92,93]. An experimental study [94] indicated that the process of steam gasification that is built based on the fluidized bed reactor with/without added O_2_ can produce quantities ranging from 10–16 MJ (Nm^3^)^−1^ gas H_2_ content of 30–60 vol%. This is because there is an absence of nitrogen from air gasification in the products; additionally, there is the possibility of a homogenous WGS during the early stages of the gasification process to increase the production of hydrogen [95]. The production of hydrogen via the biomass steam gasification method is schematically presented in Figure 11.

An innovative mechanism to synthesize H_2_ without carbon dioxide or carbon monoxide fuel cells via biomass reactions, alkali metal hydroxides as well as water vapor at comparatively low temperatures (473–623 K) under atmospheric pressure was proposed by [96]. The reaction for this proposed method is as presented in Equation (27). Basic reactions for the gasification process for biomass are presented in Table 2.

(27) $$C_{6}H_{12}O_{6}+12\mathrm{NaOH}+H_{2}O\to 6{\mathrm{Na}}_{2}{\mathrm{CO}}_{3\;}+12H_{2}$$

##### Hydrogen Production via Biomass Combustion

Combustion is basically the burning of any form of fuel to release energy in the form of heat in the presence of air. In this mechanism, biomass burns in a furnace or boiler directly with the presence of excess air, which can be used for steam production and the resultant steam used to drive turbines, compressors, or pumps in any chemical process. Biomass has advantages as a combustion feedstock due to the high reactivity of the resulting char and the fuel, and also the high volatility of the fuel [98]. Biomass combustion is normally not ideal for the production of hydrogen because of the high formation of CO_2_ linked to it. The production of hydrogen was 9.56 vol% for algal biomass combustion [99,100]. Several other gases are emitted; some of these are NO_x_, SO_x_, CO_x_, and CH_4_, which are all dependent on the composition and source of the biomass. There is a high cost associated with the treatment of these gases, which increases the total cost of the process [99,101].

#### 3.2.3. Hydrogen Production Yield and Some Limitations Associated with the Various Biomass Processes

Despite the hydrogen production potential related to the various biomass methods presented in the earlier section, the various techniques have some limitations which can hinder their use for the generation of hydrogen. Some of these are highlighted below:

➢ Biological process:
  - **Photo-fermentation**—this mechanism has low yield relative to the production of H_2_; it is also required to control the bacteria. Moreover, it requires a high surface area and has a high energy demand. The H_2_ yield is estimated to be around 9–49 g/kg feedstock [83,102].
  - **Dark fermentation**—this process require pre-treatment; additionally, it has a high number of by-products, while the rate of production and yield of H_2_ is also low. The H_2_ yield is estimated to be around 4–44 g/kg feedstock [103,104,105].
➢ Thermochemical process:
  - **Biomass pyrolysis**—this technology requires catalysts regeneration, emission of CO_2_, formation of char and tar, and variation in H_2_ as a result of complexity in biomass and variation in composition; the cost of reactor is also high. The H_2_ yield is estimated to be around 25–65 g/kg feedstock [103,106].
  - **Biomass gasification**—the limitations linked to this process are high operating temperature, variation in H_2_ as a result of complexity in biomass and variation in composition, formation of char and tar, which leads to catalyst deactivation, expensive reactor, and that CO_2_ emissions and catalyst regeneration are required. The H_2_ yield is estimated to be around 40–190 g/kg feedstock [103].
  - **Steam reforming**—this requires catalysts regeneration, operates under high temperatures, and emits CO_2_. The H_2_ yield is estimated to be around 40–130 g/kg feedstock [64].
  - **Partial oxidation**—this also operates under high temperature, includes CO_2_ emission, is adapted for only few molecules, and requires a high amount of oxygen. The H_2_ yield is estimated to be around 16–140 g/kg feedstock [107,108].

## 4. Role of Hydrogen in the World’s Future Energy Generation and Decarbonization

Hydrogen energy, as indicated earlier, has the potential to serve as an energy carrier, and this source of energy has become key in relation to global sustainable growth both in developed and developing countries [109]. Research has predicted that the world will require about 600 to 1000 EJ of primary power by 2050 [110]. Energy demands is expected to increase even more in developing countries, where the need for power is high for their development and poverty alleviation [111]. However, the current global primary energy mix is dominated by fossil fuels, which are predicted to be depleted in about 50 years using the current rate of its consumption [72,112]. Fuel cells and hydrogen are mostly seen as important technologies for a sustainable energy supply in the future. It is projected that RS shares of 36% by 2025 and 69% by 2050 on entire demand for energy could result in an 11% hydrogen shares by 2025 and 34% by 2050 [113]. Hydrogen can be transported and stored; it can be transformed into electrical energy using fuel cells. Hydrogen is ecologically friendly, contingent on the source of energy for its production; in the case of production from water, it returns to water after oxidation. A number of reasons exist as to why hydrogen is an appropriate and logical choice as a chemical fuel for the replacement of fossil fuels. The main reason is that it is a complementary energy carrier to electricity [114].

Maybe the current best-known use of hydrogen is in the transportation sector. Drivers of electric vehicles often complain about the range and time needed for recharging. Fuel cell electric vehicles that operate using hydrogen do not have such concerns, since they possess much longer range, need few behavioral changes, and have a faster refueling time. Heating of homes can also be done using hydrogen. It can be either burned alone or combined with natural gas. Proponents of hydrogen energy have suggested the use of surplus energy from wind farms at night should be harnessed to produce hydrogen, which can be stored in high pressure tanks or caverns for use [115].

The production of hydrogen could help in the reduction of curtailment in grids with a high share of variable RE electricity. Water electrolysis is a well-established industrial technology which uses electricity from the grid. It is expected to play a role on the near-period H_2_ market too [113]. It is, however, not likely to generate substantial quantity of hydrogen using wholly inexpensive or free “otherwise curtailed” electricity if, for instance, the operation of electrolyzers is only about 10% of the time or less. Considering this rate of utilization, produced hydrogen may not be competitive even if zero-cost electricity is considered. This could possibly change if the cost of electrolyzer reduces further. In order to decrease the cost of production for hydrogen, the utilization rate of electrolyzers should be higher, which is not compatible with the irregular accessibility of curtailed electricity. There is a need to strike a balance between the buying of electricity at all times of low prices and the increasing of electrolyzers utilization [116].

**Figure 12:**
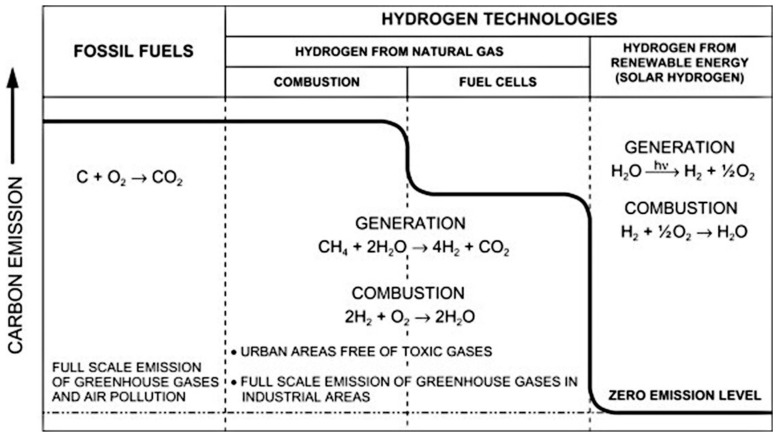
Impact of hydrogen production and combustion on emission of carbon [117]. Reproduced with permission from Elsevier, 2022.

Hydrogen is seen as a potential source of energy generation that could help lower the emission of CO_2_. A comparison among fossil fuels and hydrogen technologies is shown in Figure 12. It is estimated that the use of hydrogen generated from traditional approaches can minimize the emission of carbon by nearly 20% when used in fuel cells. Therefore, this means that emission of carbon can be significantly reduced through the production of hydrogen using RS [117]. The Hydrogen Council indicated in their report that H_2_ demand and supply could reach 10 EJ per year by the close of 2050, which is further projected to increase by approximately 5–10% per year after 2050. Therefore, H_2_ can be said to be a potential strong contender in the world’s energy system in the future [118].

### Cost Estimates for the Production of Hydrogen

The cost of obtaining hydrogen can be divided into logistics and production costs. Financial conditions such as cost of capital and local regulations also affect the final cost of hydrogen. At the production stage, the cost of the source of power, i.e., renewable power or fossil fuels, is key to the variable costs and, hence, affect the final competitiveness of every technology [119].

According to the Hydrogen Council, H_2_ was significantly used in 2020; it could potentially solve about 8% of the global energy demand (GED) and it has a production cost of about 2.50 $/kg. The cost of production has been projected to reduce to about 1.80 $/kg by 2030 to solve the GED by about 15% [120,121]. A summary of recent cost estimates for some selected technologies for the production of hydrogen is provided in Table 3.

According to IRENA [124], the use of electricity from RS to generate hydrogen could help make it competitive in terms of cost relative to fossil fuels by 2030. An amalgamation of decreasing costs of wind and solar power, economies of scale for electrolyzers, and improved performance could help in its realization.

## 5. Challenges to the Scaling-Up of Hydrogen Production

**Figure 13:**
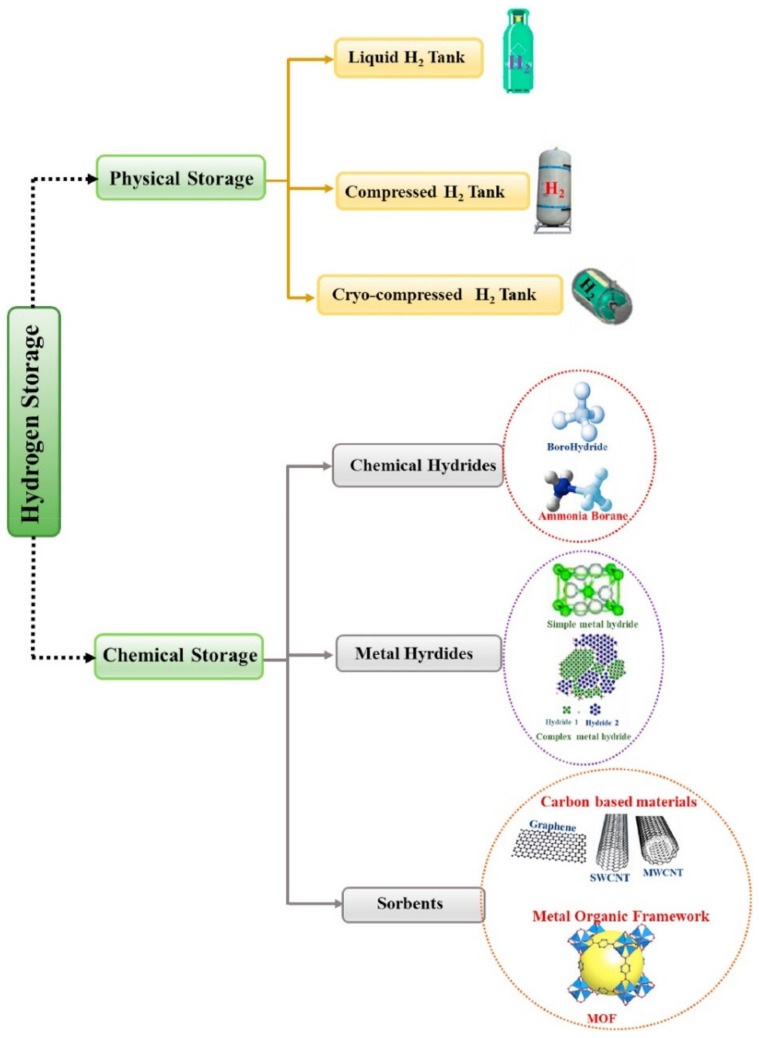
Various mechanisms for hydrogen storage [129]. Reproduced with permission from Elsevier, 2022.

This section presents some identified barriers that are hindering the production of hydrogen. It can be safe to say that the key challenges that are associated with the development of clean hydrogen is not only about the scaling up of the production of hydrogen, but also largely about the shift from carbon-intensive to low-carbon hydrogen production [125]. Some general barriers or challenges that could affect green hydrogen production are discussed below:

- Absence of a value chain for clean hydrogen—absence of existing value chain for clean hydrogen is identified as one of the major barriers in the sector that has to be overcome to help develop a low-carbon hydrogen economy. The value chain for hydrogen is currently dominated by fossil fuels; there are limited projects that focus on low-carbon hydrogen. If clean hydrogen would become globally competitive, it will require the creation of entirely new value chains. The main challenge is in relation to which path to pursue, which is particularly due to the fact that hydrogen can follow several routes in the area of demand, supply chains, and handling. It is producible, transportable, and distributable in several forms, and different sectors demand it. Therefore, the most viable outcome will rely on the infrastructures and technologies that are involved which may differ in different areas and applications. In relation to the production of hydrogen, the key challenge would be about the selection of the appropriate mechanism for its production, e.g., blue, green, or yellow hydrogen. Whereas all three mechanisms produce the same final outcome, i.e., low-carbon hydrogen, they come with dissimilar implications relative to industry, infrastructure, and most importantly effect on the environment [125].
- Storage and transportation of hydrogen—this continues to be a weak link in the energy systems of hydrogen [126]. A rise in the efficiency of these methods is linked to the resolution of two main matters: hydrogen conversion into a system that has higher density (e.g., liquefaction) and the safety improvement of conveyor systems and tanks. Additionally, while the initial matter has been studied with some hands-on solutions, problems in relation to the safe management of hydrogen has not yet been comprehensively studied [127,128]. The various techniques for storing hydrogen are presented in Figure 13. It groups the various approaches into three: (1) physical storage in liquid or pure gas forms, where there is no physical or chemical bonding with other materials, (2) adsorption, in this case the hydrogen combines with other substances through weak van der Waals bonds, (3) chemical storage, in this case other materials (e.g., chemical and metal hydrides) and the hydrogen forms chemical bonds [129].
- High production cost—the production cost for H_2_ is relatively high as a result of its immature technologies, which prevents competition with conventional hydrocarbon resources-based technologies [127]. RE-based hydrogen production mechanisms at present cannot generate hydrogen at a price that is competitive with the hydrocarbons. The high capital expenditure (CAPEX) that is required for RE-based hydrogen production is a major hindrance [130,131]. The low capacity factor for RE systems coupled with the high upfront financial burden renders it unviable with its current technologies [132]. The compression to minimize its volume for transportation bases also increases its cost, since the compression requires an advanced process. Such processes usually need expensive equipment and energy, which inflates the cost of hydrogen. Hydrogen storage in metal hydrides are possible options for their compression. Nevertheless, the metal hydrides are heavy, usually costly, and have limited lifetime, which makes such a process less practical and expensive [39,133].
- Require international standards—it is an emerging market which requires international standards and regulations, which is currently not available, which is a major hinderance to the development of a global market for hydrogen. This has led to a situation where individual countries formulate their own internal regulations and standards. The absence of common standards and regulations hinders the diffusion of hydrogen, which restrains its potentials. Formulating a common international framework is key to prevent unfair competition and free riding [125]. Areas that could gain from common standards and international harmonization include pipeline specifications and hydrogen purity for the industry, safety protocols for the sector, comparable ISO standards in the area of transportation, and guarantees of origin [134].
- Risks in investment—the hydrogen sector has risks in its supply chain as well as uncertainties on its market, which is expected to persist for a while, particularly where there are tight final product margins. Some explicit risks in the sector include creation of a monopoly in hydrogen suppliers of low-carbon hydrogen at high cost, and variations in cross-border environmental regulations. Governments are, therefore, encouraged to participate in financing projects across borders in order to help manage such risks [134].
- Flammability—the dissipation of liquid hydrogen occurs quickly due to its small molecular size, and also because it has a high diffusion coefficient. Therefore, it reduces hydrogen’s localized concentration in air and the extent of time for a potential hazard; the size of combustible cloud, however, increases. Because hydrogen has a very small energy barrier of 0.017 mJ for combustion in air, and a high flammability range of 4–74%, it tends to burn very easily with little source of ignition/spark; moreover, it is very difficult to extinguish such combusting flame [135].

## 6. Possible Strategies to Scale Up Production

Fossil-based hydrogen production technologies have a well-established feedstock infrastructure, matured production technologies, and they are also more commercialized. The technology for water electrolysis is also well matured and uses existing infrastructure and grid. These technologies are expected to play a key role in the short-term hydrogen supply market. Coal gasification and steam reforming of natural gas with carbon capture may in the mid-term continue on the hydrogen market along with biomass gasification. In the long term, water electrolysis, which uses RE electricity, thermochemical cycles, photoelectrochemical, and biological methods, is expected to play a major role on the hydrogen market [30]. PEM electrolyzers are nearing maturity technically, and within economies of scale. Several regions, such as Europe, California, and Japan, have started its commercial deployment. The objective is to use existing infrastructure, for instance, gas networks, and get ready for hydrogen production through RE to partially replace fossil fuel-based energy supply [116]. Furthermore, solar technologies’ efficiency is comparatively low; therefore, it is important to improve the materials used and also develop catalysis that can help improve the efficiency. Since it is also very important to produce hydrogen with minimum environmental impact, technologies such as photonic water splitting and wind-based electrolysis, which are emerging low-emission technologies, are potential technologies that can also be used in the long term [14].

It is important to institute an appropriate regulatory framework to encourage private sector investments. Such a framework could take into consideration the following [116]:

- Introduction of tariffs on long-term gas grid injection, de-risking instruments to help boost the uptake on the market to support hydrogen deployment and infrastructure, introduction of take-or-pay contracts, and schemes that allow the exemption from electricity levies and grid charges.
- Technology-neutral instruments that aims at the end-user should be adopted, for instance, mandates for RE content in the industrial sector and emission restrictions, which will trigger the demand of hydrogen in a structural manner and rationalize infrastructure investments while addressing issues that relate to carbon leakage. Financial support measures, such as tax rebates and waivers, and subsidies on capital expenditure are required to cover the initial cost premium relative to existing technologies.

## 7. Conclusions and Future Research Recommendations

Hydrogen is identified as an energy carrier that has the potential to help the world minimize its levels of GHG emissions, which are occasioned by the reliance on fossil fuels for the generation of energy. This study, therefore, presents a comprehensive review of the various technologies available for the production of clean hydrogen, limitations of the various technologies, and challenges that hinders the scaling up of the hydrogen economy. Production of hydrogen through RS offers several advantages, e.g., aside from its clean nature, the potential for a distributed hydrogen supply network model. As presented in this study, apart from the presence of the technical challenges associated with the various technologies, there are also other factors which serve as hinderances to the scaling up of the hydrogen economy. Key among these are issues relating to the absence of a value chain for clean hydrogen, storage and transportation of hydrogen, high cost of production, lack of international standards, risks in investment, and flammability.

Future studies on the various clean technologies for hydrogen should focus on the enhancement of their efficiencies. Technologies such as the use of pulsating electric fields and the use of ultrasonic fields must be researched to help the improvement of the efficiencies of RE hydrogen production. In terms of the membranes for the splitting of water, research should concentrate on polyimides, poly ether ketone, polyethylene, etc., which are economically viable. Other studies can also assess the various options that can be employed to commercialize RE-based hydrogen production; such studies can look at available infrastructure and potential market development. Further research is also required in order to understand the basics and complex stages of biohydrogen in order to help stakeholders implement it on large-scale levels. Governments are also encouraged to participate in financing projects across borders in order to help manage risks in the sector. Additionally, the high-temperature environment for biomass supercritical water gasification incurs corrosion issues and energy consumption; as a result, it is recommended to conduct further studies on the reaction thermodynamics and reaction mechanism to help obtain the optimum method that minimizes them, including the tar formed as a by-product. Government organizations, researchers, stakeholders, and policymakers are expected to use findings in this study to provide guidelines in shaping the roadmap of the hydrogen sector globally.

Future studies can also review the various materials, reactors, and industrial possibilities of the various technologies to help stakeholders know the research and development trend in the hydrogen sector.

## Figures and Tables

**Table 1:** Recent studies on the production of hydrogen.

No.	Year	Mechanism Used	Objective of Study	Major Findings/Results	Ref.
1	2017	Thermo-electrochemical production protonic membrane reformer	To obtain high-purity hydrogen within a single-stage process in an almost zero energy loss.	The study achieved a balanced thermal operating regime. A total energy efficiency more than 87% was obtained for the modeled hydrogen plant.	[42]
2	2021	Alkaline water electrolysis	To investigate the effect of electrode spacing on the production of hydrogen.	It was identified that smaller spacing distances for electrodes increases the interaction between the immersed electrode and the ionic electrolyte, which increases the rate of the electrochemical reaction, efficiency, and production of hydrogen.	[43]
3	2021	Proton exchange membrane electrolysis cell (PEMEC)	To assess the performance of PEMEC, which is operated by a photovoltaic thermal (PVT) system. It assessed the impact of thermoelectric generator (TEG) and phase change materials (PCM) on the production of hydrogen.	A combination of the PVT/TEG/PEMEC system performed better than other systems. The PVT/PEMEC/PCM system recorded negligible effect.	[44]
4	2019	Evaluation of solar driven natural gas reforming system	To assess the impact of a combination of steam methane reforming with carbon dioxide as well as steam-based autothermal reforming.	There was an improvement in both exergy and energy efficiencies. The exergy efficiency is 31.1%, while the energy efficiency is 59.1%.	[45]
5	2021	Photo fermentation	To assess the role of catalysts in energy conversion efficiency enhancement and the yield of photo-fermentation biohydrogen from a corn stalk (CS) via strengthening the beneficial metabolic product.	The hydrogen yield was increased by 15.93% when 0.2 g/g CS of kieselguhr was added to the liquid culture.	[46]
6	2011	Photoelectrochemical	To investigate the hydrogen evolution rate for a photoelectrochemical system, which consists of platinum as a photoanode and cathode, and anodized tubular TiO_2_, solar cell, as well as seawater, which is prepared using a nanofiltration membrane.	The rate hydrogen evolution was found to be 270 mol/cm^2^ h.	[47]
7	2021	Bio-hydrogen production based on lignocellulosic biomass	To explore the syntrophic co-fermentation model for microbial community evolution evaluation and the route of carbon transfer for the co-fermentation system.	The highest level of hydrogen production is 165 mL/g with a mean hydrogen concentration of 52.3%.	[48]
8	2021	Water electrolysis, electrochemical conversion	To propose an efficient strategy to replace the oxygen evolution reaction with a partial oxidation of degradation products originating from carbohydrate.	The results indicate that there exists the potential to use industrial waste streams for sustainable hydrogen production.	[49]
9	2019	Proton exchange membrane	To propose a synthesized polybenzimidazole (PBI) composite membrane from the addition of zirconium oxide (ZrO_2_) followed with phosphoric acid.	The efficiency of the copper chloride (CuCl) electrolyzer ranged from 91–97%, which indicates that the hybrid PBI/zirconium phosphide (ZrP) membrane can serve as an alternative to the Nafion membrane.	[50]

**Table 2:** Basic reactions for the gasification process for biomass [97].

Reaction Type	Equation of the Reaction
Pyrolysis	$C_{6}H_{10}O_{5}\to 5\mathrm{CO}+5H_{2}+C$
	$C_{6}H_{10}O_{5}\to 5\mathrm{CO}+3H_{2}+{\mathrm{CH}}_{4}$
Partial oxidation	$C_{6}H_{10}O_{5}+\frac{1}{2}O_{2}\to 6\mathrm{CO}+5H_{2}$
	$C_{6}H_{10}O_{5}+O_{2}\to 5\mathrm{CO}+5H_{2}+{\mathrm{CO}}_{2}$
	$C_{6}H_{10}O_{5}+2O_{2}\to 3\mathrm{CO}+5H_{2}+3{\mathrm{CO}}_{2}$
Steam reforming	$C_{6}H_{10}O_{5}+H_{2}O\to 6\mathrm{CO}+6H_{2}$
	$C_{6}H_{10}O_{5}+3H_{2}O\to 4\mathrm{CO}+2{\mathrm{CO}}_{2}+8H_{2}$
	$C_{6}H_{10}O_{5}+7H_{2}O\to 6{\mathrm{CO}}_{2}+12H_{2}$

**Table 3:** Selected methods of hydrogen production with their cost and production efficiencies refs. [70,122,123].

Production Mechanism	Cost, $/kg	Efficiency of Process, %
Electrolysis	10.30	60–80
Thermolysis	7.98–8.40	20–45
Photolysis	8–10	0.06
Dark fermentation	2.57	60–80
Gasification	1.77–2.05	30–40
Photo fermentation	2.83	0.1
Steam reforming	2.27	74–85
Pyrolysis	1.59–1.70	35–50
Indirect bio photolysis	1.42	-
Direct bio photolysis	2.13	-
Solar thermal electrolysis	5.10–10.49	-
Solar thermolysis	7.98–8.40	-
Wind electrolysis	5.89–6.03	-
Photo-electrolysis	10.36	0.06

## Data Availability

Not applicable.

## Abbreviation

AWE	Anion exchange membranes
CAPEX	Capital expenditure
CCUS	Carbon Capture, Utilization, and Storage
DF	Dark fermentation
FP	Fast pyrolysis
GDP	Gross domestic product
GED	Global energy demand
GHG	Greenhouse gases
HER	Hydrogen evolution reaction
RS	Renewable sources
IEA	International Energy Agency
KOH	Potassium hydroxide
SOE	Solid oxide water electrolysis
SMR	Steam methane reforming
OER	Oxygen evolution reaction
PEMs	Proton exchange membranes
PNS	Purple non-sulfur
WGS	Water gas shift
ΔH	Heat of reaction
ΔGo	Free energy of the reaction
